# Why behaviour matters: Studying inter-brain coordination during child-caregiver interaction

**DOI:** 10.1016/j.dcn.2024.101384

**Published:** 2024-04-23

**Authors:** Ira Marriot Haresign, Emily A.M., Phillips, Sam V., Wass

**Affiliations:** Department of Psychology, University of East London, London, UK

**Keywords:** Inter-brain synchrony, Hyperscanning, EEG, FNIRS, Caregiver-child social interaction

## Abstract

Modern technology allows for simultaneous neuroimaging from interacting caregiver-child dyads. Whereas most analyses that examine the coordination between brain regions within an individual brain do so by measuring changes relative to observed events, studies that examine coordination between two interacting brains generally do this by measuring average intra-brain coordination across entire blocks or experimental conditions. In other words, they do not examine changes in inter-brain coordination relative to individual behavioural events. Here, we discuss the limitations of this approach. First, we present data suggesting that fine-grained temporal interdependencies in behaviour can leave residual artifact in neuroimaging data. We show how artifact can manifest as both power and (through that) phase synchrony effects in EEG and affect wavelet transform coherence in fNIRS analyses. Second, we discuss different possible mechanistic explanations of how inter-brain coordination is established and maintained. We argue that non-event-locked approaches struggle to differentiate between them. Instead, we contend that approaches which examine how interpersonal dynamics change around behavioural events have better potential for addressing possible artifactual confounds and for teasing apart the overlapping mechanisms that drive changes in inter-brain coordination.

## 1 Introduction: Inter-brain coordination in naturalistic caregiver-child interactions

Recent advancements in technology, enabling simultaneous neuroimaging of two or more interacting persons (referred to as hyperscanning), have provided researchers with novel avenues to explore the intricate dynamics between two interacting brains during social interactions (Hamilton, 2021, Hasson and Frith, 2016; Hoehl et al., 2021; Holroyd, 2022; Novembre and Iannetti, 2021; Pérez and Davis, 2023; Dumas and Fairhurst, 2021; Turk et al., 2022; Nguyen et al., 2021; Wass et al., 2020; Kingsbury and Hong, 2020).

The primary objective of most hyperscanning research is to investigate the dynamics between the brain activities of two interacting individuals during spontaneous, naturalistic interactions. These dynamics are often evaluated using various metrics for assessing inter-brain coordination, such as synchrony (commonly defined as 'when X is high, Y is high') and sequential entrainment (commonly defined as 'changes in X forward-predict changes in Y′) (Ayrolles et al., 2021; Marriott Haresign et al., 2023). In this article, we compare two distinct approaches for studying inter-brain coordination: the 'non-event-locked approach' and the 'event-locked approach'. Our aim is to show how less frequently used non-event locked approaches can provide additional insights into mechanisms underpinning inter-brain synchrony.

### 1.1 Two approaches for studying inter-brain coordination during free-flowing naturalistic interactions

#### 1.1.1 The non-event-locked approach

The primary focus of non-event locked approaches is on understanding differences in interbrain coordination across various forms, modalities, and contexts of social interaction. Inferences are then made regarding how interbrain coordination associations might facilitate successful early social interactions, based on correlations between inter-brain synchrony and behaviour (Nguyen et al., 2023).

To achieve this, non-event-locked approaches involve examining the average levels of brain coordination across entire blocks or conditions. Typically, this process begins by calculating brain coordination within short data windows, usually lasting 1–2 seconds in EEG data (Leong et al., 2017, Santamaria et al., 2020) and approximately 30 seconds in fNIRS data (Nguyen et al., 2021). These windows are then shifted forward in time by a predetermined increment, often half the number of time points within the window, and interbrain coordination is recalculated. This iterative process generates a single value per window, which is subsequently averaged across all windows to provide a comprehensive representation of interbrain coordination throughout the dataset. Average interbrain coordination values are typically compared between experimental conditions (e.g., Leong et al., 2017; Nguyen et al., 2021; Piazza et al., 2020; Santamaria et al., 2020) and/or correlated with outcome measures (Davidesco et al., 2023, Nguyen et al., 2020). For instance, linear regression-based analyses can be employed to track relationships between interbrain coordination and behaviour (Djalovski et al., 2021; Pérez et al., 2017; Dikker et al., 2017). Alternatively, indirect approaches involve regressing out behaviour from individual brain activity patterns and recalculating interbrain coordination, often utilizing techniques like General Linear Modeling (GLM) (e.g., Pinti et al., 2020). For example, Piazza and colleagues (2020) demonstrated that regressing out continuous behavioural time series (mutual gaze, infant smiling, and joint object attention) from caregiver and infant individual patterns of prefrontal cortex fNIRS activity resulted in decreased caregiver-infant interbrain coordination.

As this approach does not primarily aim to determine the presence or absence of interbrain coordination within the data, statistical procedures to test the observed interbrain coordination against chance are often not applied. Instead, the focus is on comparing effects between individuals or conditions. While it is possible to conduct analyses comparing whether interbrain coordination during non-event-locked analyses significantly differs from chance (e.g., Astolfi et al., 2010a; 2010b; 2011; Babiloni et al., 2007, Babiloni et al., 2007; Kawasaki et al., 2013; Yun et al., 2008; Dikker et al., 2017; Goldstein et al., 2018; Hu et al., 2018; Kinreich et al., 2017; Endevelt-Shapira et al., 2021), these are often not applied.

#### 1.1.2 The event-locked approach

The second approach, termed the event-locked approach, assesses changes in interbrain coordination relative to individual behavioural events (e.g., onsets of specific behaviours). This approach, common in intra-brain coordination literature, involves event-locking brain activity to specific behaviours produced by individuals or externally presented events, enabling detailed examination of temporal dynamics in the associations between neural activity across different brain regions (Friston, 1994, Friston et al., 2003, Honey et al., 2009, Horwitz, 2003, Horwitz and Glabus, 2005, Kahan and Foltynie, 2013, Sporns, 2007). Statistical tests are employed to compare changes in interbrain coordination relative to chance, around specific behavioural events, in order to determine significant patterns of interbrain coordination and their temporal scales (e.g., Lindenberger et al., 2009; Gugnowska et al., 2022; Marriott Haresign et al., 2023). Statistical testing most often utilizes standardized, permutation-based clustering measures of EEG activity (Maris and Oostenveld, 2007).

In this article, we build on previous work (Marriott Haresign et al., 2022; Bilek et al., 2022) to argue against the exclusive use of non-event-locked approaches, which currently dominate naturalistic developmental interbrain studies. Instead, we advocate for complementary, event-locked, approaches akin to techniques used in intra-brain coordination studies. We present arguments on both practical and theoretical fronts. Firstly, in Section 2, we argue that non-event-locked methods can produce highly reproducible results but are vulnerable to the possibility that the differences observed are due to artifact - i.e., signals in neural data that are not generated by the brain, but instead through EMG activity induced through movement. Secondly, in Section 3, we contend that relying solely on non-event-locked approaches hinders the development of a comprehensive understanding of the how interbrain coordination is achieved (see Marriott Haresign et al., 2022).

## 2 Artifact and event-locked naturalistic analyses

In this section we focus on practical issues associated with studying inter-brain coordination during naturalistic dyadic interactions, before we go on to consider theoretical issues in Section 3. First, we consider artifact in EEG (Section 2.1). We provide a worked example by considering how eye movement artifact might contribute to observed reports of inter-brain coordination in EEG studies (Section 2.2). Then, we examine artifact in fNIRS (Section 2.3). We provide a worked example of how respiration artifact might contribute to the false impression of inter-brain coordination in fNIRS studies (Section 2.4). Finally, we discuss how the use of event-locked methods in naturalistic dual-brain neuroimaging studies can mitigate some of these problems (Section 2.5). Throughout this discussion, we define artifact as any signal in the EEG or fNIRS data that is generated outside of the brain. A typical example is that during blinks, the eyelids slide across the positively charged corneas, allowing current to flow to the forehead. This is readily observed in frontal EEG electrodes (Matsuo et al., 1975; Dimigen, 2020).

### 2.1 How movement during naturalistic interaction paradigms creates artifact in EEG data

In naturalistic interaction paradigms, participants do not sit still. Even when movement is constrained (e.g., by sitting the child in a highchair), children are moving their eyes typically several times per second. They also move their torsos, heads, limbs and faces; and they vocalise. It is important to note that many of these problems are also observed in traditional screen paradigms, where, for example, eye movements occur systematically, time-locked to the appearance of new objects on-screen (e.g., Dimigen et al., 2009, Dimigen et al., 2011; Yuval-Greenberg et al., 2008), and participants can become fidgety during repetitious screen paradigms (Marriott Haresign et al., 2023). But our discussion here will concentrate on naturalistic paradigms.

**Fig. 1:**
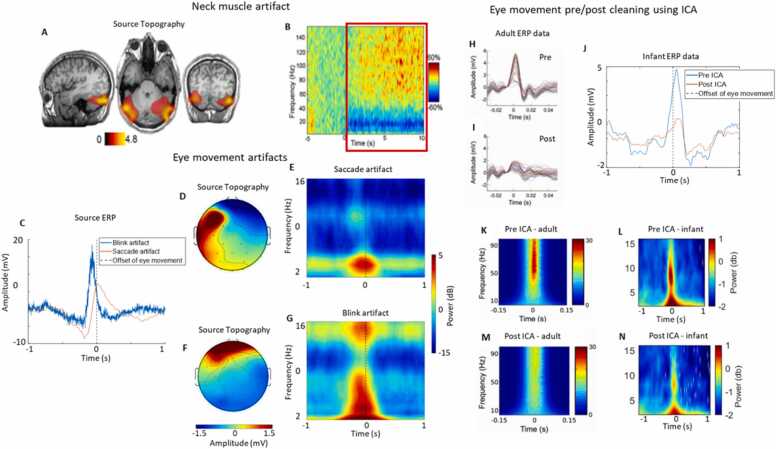
Movement-generated artifact contaminates the EEG with varying topographical and spectral features and cannot be readily removed. A-B) Example muscle artifact in adult MEG data whilst participants were using a joystick - recreated from Muthukumaraswamy, 2013. A) Difference in source MEG power whilst using the joystick. B) Time-frequency activity whilst using a joystick (time 0 is the onset of movement) – red box highlights contaminated section of data. This activity is likely reflecting the increased postural activity of upper neck muscles during movement of the joystick. C-G) Example of ocular artifact in infant EEG data relative to spontaneous eye movements during social interactions. C) Sample source ERPs of two overlapping eye movement artifacts. D) Sample source topography of blink artifact. E) Sample source time-frequency power of blink artifact. F) Sample source topography of saccade artifact. G) Sample source time-frequency power of saccade artifact. H-J) Example of the effectiveness of ICA for cleaning eye movement artifact from infant and adult ERP data. H) Frontal scalp ERP time-locked (time 0) to the offset of saccadic eye movement before ICA cleaning in adult EEG data. I) Same adult data after ICA cleaning. ICA cleaning reduced artifact to about one third of the original amplitude. Recreated from Plöchl et al, (2012). J) Frontal scalp ERP time-locked to offset of saccadic eye movement before and after ICA cleaning (iMARA) in infant EEG data. K-N) Time-frequency representation of the effectiveness of ICA for cleaning muscle artifact. K) Frontal scalp time-frequency power relative to offset of saccadic eye movement before ICA cleaning in adult EEG data. L) Frontal scalp time-frequency power relative to offset of saccadic eye movement before ICA cleaning in infant EEG data. M) Frontal scalp time-frequency power relative to offset of saccadic eye movement after ICA cleaning in adult EEG data. N) Frontal scalp time-frequency power relative to offset of saccadic eye movement after ICA cleaning in infant EEG data. A very similar reduction of artifact was observed in the nosier infant EEG data. Given that data is very likely still to contain residual artifact consideration of behaviour is essential, particularly in situations in which movement covaries with cognitive function.

It is well established that the spectral range of muscle activity (∼20–300 Hz) overlaps with high-frequency neural activity (Criswell, 2010; see Muthukumaraswamy, 2013 for a review), and that even state-of-the-art cleaning techniques are unable to remove this artifact fully. Because of this, many developmental scientists typically concentrate on lower-frequency neural dynamics (Marshall et al., 2011, Leong et al., 2017; Xie et al., 2018; van der Velde et al., 2019; Jones et al., 2020) and apply low pass filters during artifact rejection (e.g., at ∼30 Hz in adults (Niedermeyer and da Silva, 2005; Fries et al., 2008) and ∼20 Hz in infants (Wass et al., 2018; Marriott Haresign et al., 2023). Several studies have, though, illustrated muscle-related artifact contamination at much lower frequencies (Whitham et al., 2007). For example, infant motion (jaw movements, limb movements of the hand, arm, foot, and leg) leads to increases in beta (∼15 Hz) power and trending effects of decreased power in infant theta (3–6 Hz) and alpha (6–9 Hz) power (Georgieva et al., 2020). Additionally, research has shown, consistent with the adult literature (Dimigen et al., 2009, 2020; Plöchl et al., 2012), that eye movement artifact can only be partially removed from visually evoked potentials, even using sophisticated machine learning ICA-based artifact rejection techniques (Marriott Haresign et al., 2021) (see Fig. 1).

### 2.2 How eye gaze artifact might create the impression of inter-brain coordination in EEG hyperscanning data

The existence of residual artifact in EEG recordings is relevant for any analyses that examine the neural correlates of visual processing. For example, studies that examine individual differences in resting theta power while infants view dynamic video materials on-screen (e.g., Wolfe and Bell, 2007; Tomalski et al., 2013; Henderson et al., 2004; Begus et al., 2020; Haartsen et al., 2022) may be influenced by individual differences in how often infants move their eyes while viewing the material influence between-condition comparisons. But how is it relevant for dual-brain analyses? In this section, we outline how EEG artifacts generated by eye movements might limit our ability to interpret inter-brain coordination analyses.

**Fig. 2:**
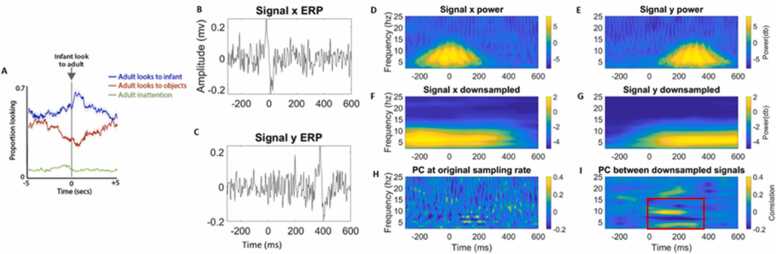
Illustration that coordination during social interaction often results from lagged contingent responding and that neural activity both precedes and follows these behaviours. A) Probability of changes in adults gaze peaks around ∼1 second after changes in infant gaze. B) Example ERP plot of signal ‘x’. C) Example ERP plot of signal ‘y’. D) Time-frequency power of signal x. E) Time-frequency power of signal y. F) Down sampled, time-frequency power of signal x. G) Down sampled, time-frequency power of signal y. H) Spearman’s correlation of single trial power (PC) between x and y) computed at each time-frequency point (i.e., original temporal scale of data). I) Spearman’s correlation of single trial power between x and y computed on the down sampled data. The AOIs on panel f indicate regions of significant correlations. D-I from Marriott Haresign et al., (2022).

During social interaction, eye movements are not random: they are influenced by the interacting partner’s behaviour, and the ongoing, bidirectional dynamics of the exchange (Valtakari et al., 2021). For example, Fig. 2A shows adults’ gaze behaviour time-locked to instances where the infant looks up to the adult during a face-to-face interaction. An increase in the likelihood of the adult looking at the infant is observed in the 1-second window following the infant gaze shift. Based on the argument given above, we know that this will lead to eye movement artifact in the infant’s data, followed by eye movement artifact in the adult’s data shortly afterwards (see Fig. 1 for examples of eye movement artifact). In addition, it will lead to genuine neural activity as both infants and adults respond to changes in the others’ gaze.

We know from previous research that eye movement artifact and neural responses to gaze tend to overlap in time and frequency (e.g., Marriott Haresign et al., 2021, see also Fig. 7). Fig. 2B-2I, which are based on simulated data, illustrate how temporally lagged neural responses could give rise to the impression of inter-brain coordination. However, this relationship could just have easily been generated by eye movement-induced artifact, which often closely resembles ERPs (see Fig. 7) and manifests both in the time (Fig. 1J) and frequency domains (Fig. 1L). Non-event-locked analyses do not examine the temporal dynamics of inter-brain coordination relative to behavioural events. Therefore, it is challenging to infer whether the observed inter-brain coordination is the result of the neural responses to changes in gaze or the result of the eye movement artifact.

A final point is that differences in amplitude and power can also create the impression of differences in phase. Although power and phase estimations are thought to be independent, in reality they are often very difficult to separate (Burgess, 2013). This is relevant when interpreting changes in inter-brain coordination, as there is still uncertainty over what combination of evoked and induced responses generate ERPs (Burgess, 2012). Briefly, evoked responses are additive signals superimposed upon the background/ongoing EEG, whereas induced responses are changes in power and/or phase that take place within the background/ongoing EEG. As shown in Fig. 1, movement-related artifact leads to increases in the spectral power of EEG data (Fig. 1B, E and G). Changes in spectral power resulting from movement-induced artifact can give the appearance of increased phase locking/ resetting, due to changes in signal-to-noise ratios and errors associated with estimating phase (Muthukumaraswamy et al., 2011).

**Fig. 3:**
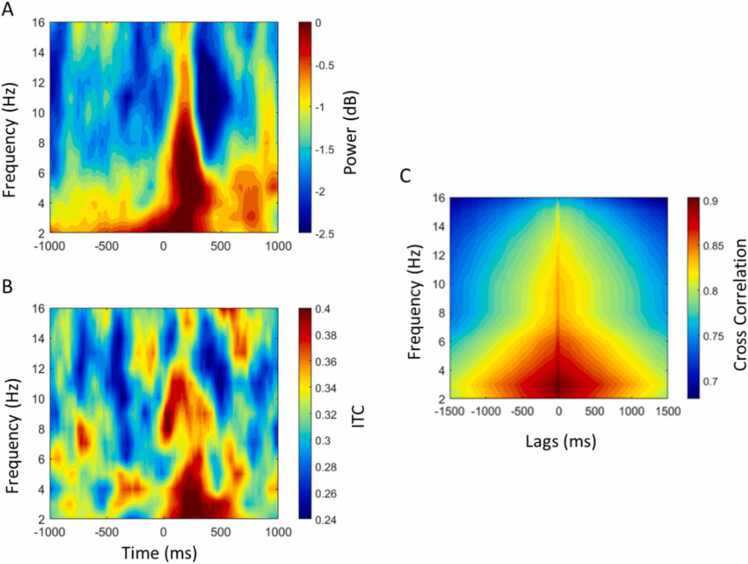
Illustration of the relationship between changes in intra-brain power and phase and inter-brain phase locking. A) Occipital scalp time-frequency power relative to the offset of saccadic eye movement in infant EEG data. B) Occipital scalp time-frequency inter-trail coherence (ITC) relative to offset of saccadic eye movement in infant EEG data. C) Cross-correlation between infant occipital time-frequency power and caregiver-infant inter-brain phase locking (occipital). 1 lag = 20 ms.

Fig. 3 shows changes in spectral power (Fig. 3A) and phase locking (Fig. 3B) that are confounded by eye movement artifact in infant EEG data. Note the overlapping nature of these signals. We also show how this can impact changes in inter-brain coordination; Fig. 3C shows a cross-correlation between infant EEG power and caregiver-infant inter-brain coordination. Thus, increases in spectral power resulting from eye movement-related artifact are likely to heavily confound measures of inter-brain coordination, if not controlled for. As shown in Fig. 3, event-locked methods provide an easy way to visualize this relationship. Using non-event-locked methods that average large amounts of data it would be very difficult to track and quantify relationships between power and phase and how this might confound measures of inter-brain coordination.

### 2.3 How movement during naturalistic interaction paradigms creates artifact in fNIRS data

**Fig. 4:**
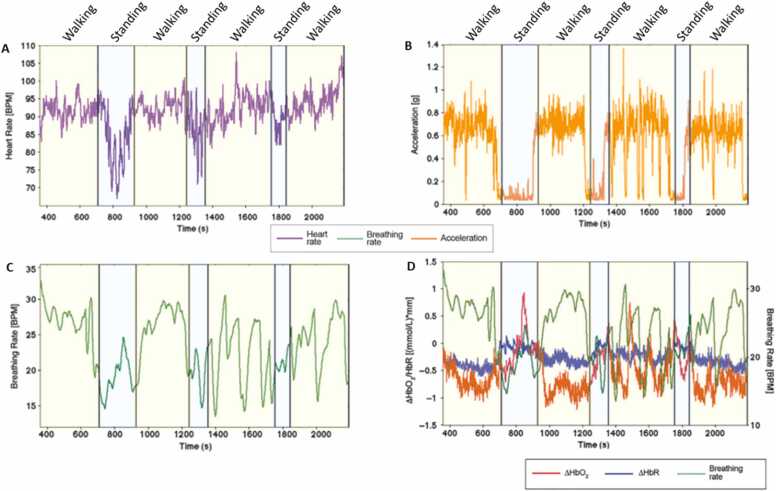
Illustration of the impact of walking on raw fNIRS data. Examples of heart rate (A), acceleration data (B) and breathing rate (C) from one participant during periods both when the participant is walking (yellow shaded areas, labelled ‘W’) and standing (blue shaded areas, labelled ‘S’). D) Example fNIRS data illustrating changes during walking vs standing, that covary with changes in breathing rate. The differences in the fNIRS signal between walking and standing could be attributed to cognitive function based solely on the fNIRS data, but by simultaneously tracking behaviour it becomes clear that these changes are a result of physical exertion. Figure recreated with permission from Pinti et al., (2018).

Although it is typically assumed to be less susceptible to artifact (Pinti et al., 2018), recent research has shown that fNIRS data is susceptible to different types of artifact induced both by physiological changes and by small (e.g., movement of fingers) and large (e.g., movement of legs) bodily movements (Cockx et al., 2023). Motion-induced fNIRS artifact arises from increases in blood volume. For example, Pinti and colleagues (2018) observed changes in the concentration of oxy- and deoxyhaemoglobin coupled with increases and decreases in both heart rate and breathing rate levels at times when participants were walking vs standing (Pinti et al., 2018). This suggests that the most clearly observable differences in neural activation between walking and standing were not a result of differences in cognitive function but rather a result of differences in cardiac output. Further, changes in breathing rate exhibit trends very similar to concentration signals, and in particular changes in oxyhaemoglobin (ΔHbO2) (Kirilina et al., 2012, Tachtsidis and Scholkmann, 2016), both when the participant is walking (see Fig. 4, yellow shaded areas) and standing (blue shaded areas). Other recent research has also shown that facial movements such as frowning can introduce artifact in optodes over the forehead (Yücel et al., 2014).

Just as with EEG data, however, many fNIRS inter-brain coordination studies do not currently track face movements, and physiological measures such as blood pressure, heart rate, and breathing. Doing so would lead to a better understanding of how movement-related artifacts contribute to fluctuations in concentration signals in both interacting partners (Metz et al., 2017; Scholkmann et al., 2017; von Lühmann et al., 2019, 2020).

### 2.4 How respiration artifact may create the impression of inter-brain coordination in fNIRS hyperscanning data

Speech elicits changes in respiratory patterns compared to rest (Hoit and Lohmeier, 2000) and is a powerful modulator of cardiovascular variability (Pinna et al., 2006). Respiration directly influences hemodynamic responses that are detected by the fNIRS signal (Tachtsidis and Scholkmann, 2016). For example, Beda and colleagues (2007) argued that interindividual differences and rest–task changes in HRV and SAPV in the low-frequency band (0.04–0.15 Hz) can be explained by variations in the respiratory volume signal (Beda et al., 2007).

**Fig. 5:**
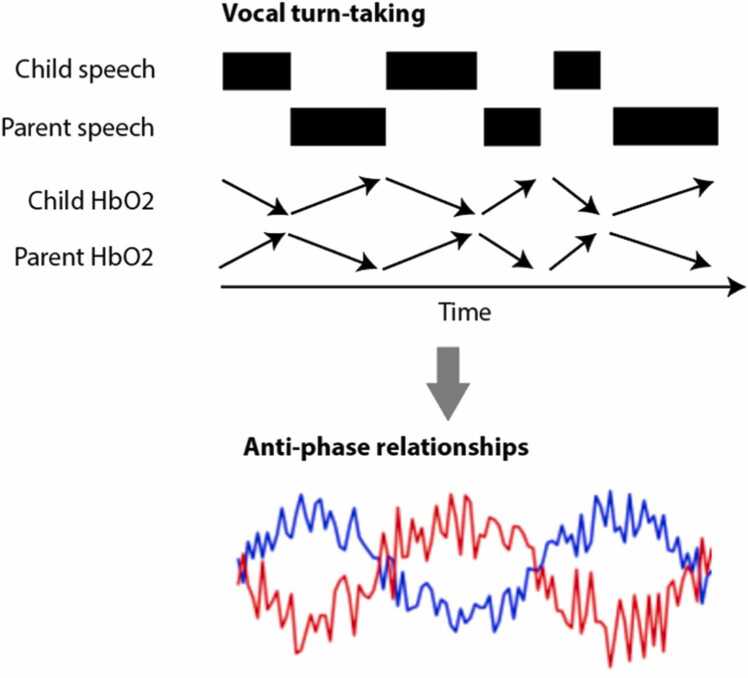
– schematic showing how turn-taking might lead to anti-phase associations in HbO2 which could give the appearance of inter-brain synchrony.

It is well established that, during social interaction, vocal turn-taking develops (Jaffe et al., 2001; Gratier et al., 2015), one partner speaks while the other is silent, and *vice versa*. This pattern will, naturally, create a profile in which hemodynamic decreases are taking place in one partner (while they are speaking), at the same time as hemodynamic increases are taking place in the other partner (while they are resting) (as illustrated in Fig. 5). Potentially, this could create anti-phase relationships, which might manifest as concurrent inter-brain coordination of hemodynamic changes in many of the measures (such as wavelet transform coherence) that are widely used to index concurrent synchrony in dual-brain fNIRS studies. In using non-event-locked analyses, it would be challenging to separate out hemodynamic changes associated with differential patterns of breathing during speaking/listening from cognitive processes associated with auditory perception of another’s speech and the planning and production of one’s own speech. Event-locked approaches might help here. Looking, for example, at changes in inter-brain coordination relative to the onsets and offsets of speech might add further insights into how different drivers are contributing to inter-brain coordination. For example, there could be hemodynamic changes associated with the brain predicting when to next speak which would have a different but potentially overlapping timescales to hemodynamic changes associated with breathing during speaking.

### 2.5 Addressing artifact in naturalistic studies: analysing and comparing event-locked changes in EEG and fNIRS

How might future research analyse inter-brain coordination in light of potential confounding artifacts induced through movement? In this section, we argue that if behaviours are recorded in detail, and at a sufficiently fine-grained temporal resolution, it is possible to design analyses that get around the problem of residual artifact in the data.

Firstly, using non-event-locked methods, one solution is merely to measure the behavioural variables of interest, which may be influencing the neuroimaging data, and either include them as covariates or include separate analyses to examine how behaviours differ between conditions. Some measures can be extracted relatively easily from video recordings of interactions using standard machine learning packages, such as limb and hand position (OpenPose, Cao et al., 2017), facial feature movement (FaceAlignment, Zhu et al., 2016) and respiration (Janssen et al., 2015). For physiological measures such as respiration and heart rate, physiological monitors will provide greater accuracy at low cost. Gaze direction can be measured using an eye-tracker or, less invasively, using either a Kinect sensor (Mora and Odobez, 2012) or hand-coding gaze direction (although both approaches will be too low-resolution to detect small-scale eye movements).

**Fig. 6:**
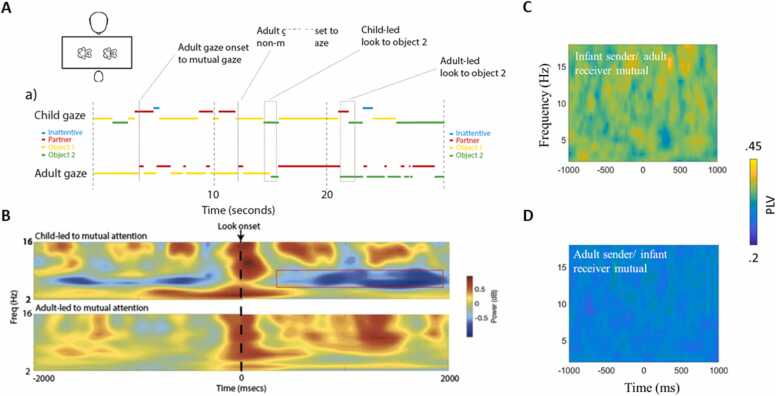
A) Example of a 30-second segment of gaze data from a tabletop caregiver-child interaction paradigm. B) Example of an analysis (showing power changes in the infant, not inter-brain coordination) that can be applied to compare two types of gaze shifts in naturalistic data. Both lead to identifiable residual artifact in the EEG (observable broadband around Time 0); but power differences before and after the gaze shift can be examined nevertheless (Phillips et al., 2023). C) Inter-brain coordination relative to infant sender/adult receiver mutual gaze onsets. C) Inter-brain synchrony relative to adult sender/infant receiver mutual gaze onsets (from Marriott Haresign et al., 2023).

Using this approach, we can examine changes in inter-brain coordination relative to specific observed events. For example, we can event-lock our data to moments where the adult looks at the child (see Fig. 6A) and compare moments where the adult gaze shift happens when the child is already looking at the adult with moments where the adult gaze shift happens when the child is looking elsewhere (Fig. 6B). In this way, the adult’s (or infant’s) eye gaze artifact is equivalent between the two conditions, and the only difference is whether the gaze shift leads to mutual gaze or not. We can also examine directed changes in inter-brain coordination that are time-locked to an eye movement. Fig. 6C and D show caregiver-infant interbrain coordination time-locked to infant and adult-initiated mutual attention. Here, we can see that, despite changes in eye gaze leading to a pronounced artifact in the time-frequency domain in both adult and infant EEG activity (Fig. 6B), this does not result in changes in caregiver-child inter-brain coordination. Here we show how using event-locked methods provides a clear way of examining the extent to which movement-induced artifact is impacting inter-brain coordination.

## 3 Studying behaviours that drive inter-brain coordination

In this next section, we consider theoretical perspectives on what drives inter-brain coordination during social interactions. Here, we outline our arguments for why event-locked approaches are crucial for developing a mechanistic understanding of how inter-brain coordination is established and maintained during a free-flowing interaction.

**Fig. 7:**
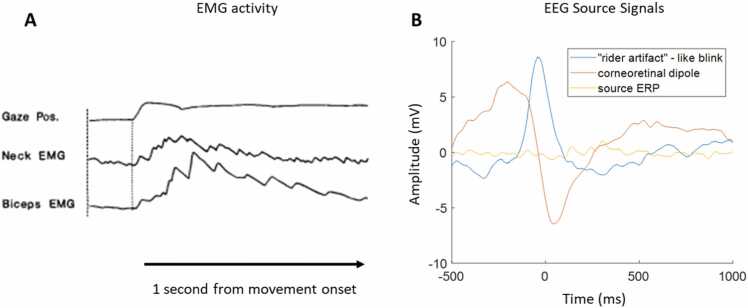
Illustration of multimodal overlapping nature of human behaviour. A) Sample changes in gaze position, neck and bicep EMG relative to experimentally guided reaching behaviours, from Biguer et al., 1982, with permission. Sample source ERPs time-locked to the offset of a saccadic eye movement derived from ICA. Both plots illustrate that for a given behaviour e.g., reaching or looking, multiple muscular and cognitive systems are activated. Using additional sensor (A) or source separation techniques (B) it is possible to examine the contribution of these different sources of activity towards interpersonal behavioural coordination.

The core problem with non-event-locked methods is that it is challenging to link individual behavioural or environmental events with changes in brain activity. One way to do this is to try to isolate where in the brain the observed effects are generated, assuming, based on previous literature, that certain brain areas ‘perform’ certain cognitive functions/ behaviours. In reality, however, observed effects are caused by a cascading and overlapping mix of systems (e.g., see Fig. 7) (Pessoa, 2023).

**Fig. 8:**
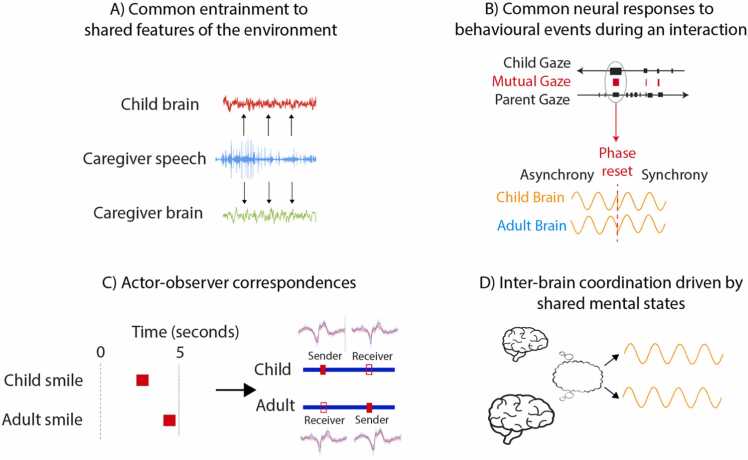
Schematic illustrating the four potential drivers of inter-brain coordination that we discuss in Section 3.

In the following sections, we describe how event-locked methods can be used to study how separate and overlapping behaviours give rise to behavioural coordination in order to more accurately disentangle how inter-brain coordination is achieved and maintained. In the sections that follow (3.1–3.4) we discuss four potential drivers of inter-brain coordination: common entrainment to shared features of the environment (section 3.1); concomitant inter-brain synchrony to interaction behaviours (section 3.2); sequential synchrony to actor-observer relationships (section 3.3); and synchrony induced through higher-order cognitive processing (section 3.4) (see Fig. 8). These potential drivers span different levels (e.g., behavioural and cognitive) and modalities (e.g., changes in spectral power or phase) and are not mutually exclusive.

### 3.1 Common entrainment to shared features of the environment

The presentation of a common external stimulus evokes highly similar neural responses across many individuals. For example, research has shown that the infant brain synchronises with features of the external environment, such as speech and visual information (Jessen et al., 2019). Given that intra-individual neural responses are typically very similar, even in the absence of interaction between two individuals, it would be possible to observe associations in their neural activity, in cases where they were presented with the same external stimulus (Hasson et al., 2008, Kauppi et al., 2010, Nastase et al., 2019). Aligned patterns of neural activity in two interacting brains could therefore arise through intra-individual coordination to a shared external stimulus (e.g., the caregivers’ speech signal) (Fig. 8a). This is something that researchers have cautioned about interfering with measures/ understanding of inter-brain coordination (Burgess, 2013, Holroyd, 2022) and in many cases researchers take steps to control for this in their experimental designs and analyses (by contrasting experimental conditions with similar external stimulus, e.g., Gugnowska et at., 2022).

Importantly, however, there are several ways in which concomitant inter-brain coordination driven by an external stimulus could be important to understanding the neural mechanisms that support successful social interactions. The first is to look at whether this type of coordination is subject to cognitive influences (Simony et al., 2016). To address this, we could conduct windowed analyses to examine associations between the phasic variation of different properties of the external stimulus and environment-brain and inter-brain coordination (e.g., Hendrikse et al., 2023). For example, Perez and colleagues (2019) compared inter-brain coordination while speaking and listening to speech in a native vs foreign language.

There are a number of ways in which future research can improve our understanding of this driver of inter-brain coordination. For example, does inter-brain coordination peak around individual words or linguistic units supporting speech processing, as has been shown for individual brain-to-speech coordination (Doelling et al., 2014)? Does it peak around behavioural events, such as the onset of mutual gaze (Çetinçelik et al., 2023)? And/or does it change as a function of different interpersonal social mechanisms – such as familiarity with the speaker’s voice, or the ability to form predictions about the upcoming content, and so on? In this example event-locking inter-brain coordination to the onsets of words, sentences, rises in the speaker’s pitch and behavioural events such as mutual gaze onsets provide a natural way to provide additional information about how inter-brain coordination is established and maintained during social interaction.

### 3.2 Common neural responses to behavioural events during an interaction

Several researchers have suggested that inter-brain coordination could be driven by both partners concomitantly synchronising their neural activity to behavioural events during an interaction (Wass et al., 2020, Leong et al., 2017). For example, it has been suggested that inter-brain coordination may increase within a dyad following communicative signals (such as gaze, gestures, or vocalisations) that cause a concurrent phase reset (i.e., an abrupt shift in the cycle of underlying oscillatory activity) in both interacting partners (Fig. 8b). Here, neural oscillations in both the sender (of the social signal) and the receiver’s brain, that were previously random with respect to each other (low inter-brain coordination), would be simultaneously reset in response to a communicative signal. Following this reset, the neural activity of both the sender and the receiver would oscillate with more consistent variation over time (high inter-brain coordination).

Several studies looking at different aspects of inter-brain coordination have reported that on average, inter-brain coordination is higher during periods of mutual gaze compared with non-mutual gaze in adult-child dyads (Leong et al., 2017) and adult-adult dyads (Kinreich et al., 2017, Luft et al., 2022) – although our own work which aimed to use identical methods to the Leong study failed to replicate these earlier findings (Marriott Haresign et al., 2023). But even if it is true that inter-brain coordination is higher during mutual gaze, is this because inter-brain coordination is associated with mutual gaze a result of both individuals’ brains concomitantly responding to the *onset* of mutual gaze? Or does it emerge during the course of mutual gaze, and if so why? Answering this question appears vital to understanding the mechanisms through which inter-brain coordination is achieved and maintained (Marriott Haresign et al., 2022). It is only if the former possibility is true, and inter-brain coordination is highest during the periods immediately following the onset of mutual gaze, that the account shown in Fig. 8b might be correct (Marriott Haresign et al., 2022). At the moment, the available evidence does not support this (Marriott Haresign et al., 2023).

### 3.3 Actor-observer correspondences

The third mechanistic account of how inter-brain coordination arises during social interaction is the idea that, during shared interactions, similar but overlapping patterns of brain activity occur as a result of each individual controlling their own motor behaviours, and at the same time responding to the motor behaviours of their partner (Hamilton, 2021, Kingsbury et al., 2019) (Fig. 8c). For example, using non-event-locked analyses, Kingsbury and colleagues employed microendoscopic calcium imaging (which has a relatively low temporal resolution of ∼1 sec) to record from the dorsomedial prefrontal cortex (dmPFC) of interacting mice during social interactions (Kingsbury et al., 2019). Data were analysed by adding behavioural data and cross-brain data to a traditional general linear model (GLM) to model the activity of one individual’s brain in terms of both their behaviour and their partner’s behaviour and brain activity (cross-brain GLM or xGLM). They identified populations of neurons that encoded the mouse’s own actions and the actions of the partner mouse. The summed activity across the dmPFC showed coherence across the two animals that disappeared when these self/other coding neurons were removed (Kingsbury et al., 2019).

However, we know that actor-observer correspondences are not one singular dynamic but unfold over multiple timescales. For example, Richardson and colleagues (2007) showed that temporal associations in gaze between interacting adult-adult dyads were strongest at lags of around 2 s, meaning that changes in the spatial focus of one person’s attention led to changes in another person’s attention 2 seconds later. Further Hale and colleagues (2020) measured coordination in head movements between adults engaged in structured conversations. Their aim was to investigate whether coordination was best modelled by concurrent synchronisation (e.g., no time lag), rapid and reactive mimicry (with short lags under 1 s) or traditionally observed mimicry (with longer lags of several seconds). Evidence suggested that this coordination was generated by a mechanism with a constant lag of 600 ms between the leader and the follower consistent with the rapid model of mimicry. Delineating the different timescales underlying actor-observer correspondences cannot be understood using non-event-locked methods, but rather necessitates event-locked approaches.

For example, in Fig. 2 we show how contingent responsiveness in gaze, depending on the analytical approach, might be measured as either a concurrent zero-lagged synchronous relationship or a Granger-causal temporally-lagged synchronous relationship. Fig. 2A shows contingent responsiveness in caregiver-infant gaze. The probability that the adult will look towards the infant increases in the 1 second following the infant’s gaze to the adult. Fig. 2B and C show simulated neural responses to the changes in gaze. Importantly they show a temporally lagged relationship – signal y (Fig. 2C ‘adult’) changes later than signal x (Fig. 2B ‘infant’). These signals both show a low frequency (<15 Hz) increase in spectral power (Fig. 2D and E). When inter-brain coordination is calculated at the original time scale of the data and is not averaged, very little inter-brain coordination is observed (Fig. 2H). However, because non-event-locked approaches can involve heavy down-sampling of the data prior to/ during the calculation of inter-brain coordination, this can create the impression that two events that occur slightly after one another in time are actually occurring at the same time. Fig. 2F and G show the same spectral power (as in D and E) that has been spread out due to averaging/ smearing which creates a temporal overall between signals x and y. The result of this can be measured as an artificial increase in concurrent inter-brain coordination (Fig. 2I).

Another potential contribution that event-locked methods can make here is to examine differences between types of contingent responsiveness – comparing fast automatic processes with slower and more effortful behaviours. These different routes may operate through quite distinct neural mechanisms (e.g., Hale et al., 2020; Heyes et al., 2021). In event-locking measures of brain activity to fluctuations in each individual’s behaviour it is possible, for example, to distinguish more automatic processes such as mimicry from the more controlled operations involved in mutual anticipation. For example, using both regression-based analyses that regress individual behaviours and composites onto the brain data, and/or event-locked analyses, we can examine whether changes in the observer’s brain activity precede the actors. If simple mimicry is occurring, then this might be modelled as a Granger-predictive relationship between behaviour-associated brain activity in partner 1 (the actor) and partner 2 (the observer). Here event-locked analyses should show activity in the observer time-locked to after the onset of the actor’s action (see e.g., Southgate et al., 2009). If response anticipation and preparation is occurring, then analyses should show temporally co-occurring activity in the observer and the actor. As we show in (Fig. 2), only a fine-grained temporal resolution will allow us to study whether actors are responding to one another or anticipating; using non-event-locked methods will present challenges in our ability to distinguish this.

### 3.4 Inter-brain coordination driven by shared mental states

At the moment, we understand relatively little about how action-generated contingencies drive higher-order states during early caregiver-child interaction. For example, some work has suggested that higher-order mental states drive interpersonal coordination in adults (e.g., Goupil et al., 2021) and that this can drive interpersonal coordination even without behavioural input. For example, Wel and Fu (2015) examined behavioural coordination within a joint action paradigm. Participants sat next to a confederate while simultaneously moving their right hand back and forth between two targets in time with the beat of a metronome. The researchers manipulated the timing of the metronome beats (either presenting them as a continuous stream (one every 850 ms) or in discrete pulses (two beats presented, followed by 2-second pause)), the trajectory of the confederate’s hand movements (by placing an object between the two targets requiring the confederate to raise their hand over the object to reach the target) and whether or not dyads could see each other’s actions or not (though in the no vision condition participants initially observed whether or not the confederate needed to clear the object or not). The authors observed that when confederates modulated the height of their hand movements in order to clear the object, participants also modulated the height of their hand movements. This effect was irrespective of whether or not participants could see the confederate’s actions or not. This suggests that behavioural coordination (joint action) was driven not by visually inputted sensorimotor information but rather by higher-order mental processes such as participants’ shared task states – which emerges when an individual shares another individual’s mental states, e.g., the knowledge that certain events have given outcomes.

To what extent do shared mental states drive inter-brain coordination in early caregiver-child interactions beyond sensorimotor perception-action coupling (Fig. 8d)? And to what extent does this depend on actor-observer correspondences? For example, some research has suggested that gamma band activity is associated with mentalising processes driven by nonverbal social cues in adults (Cohen et al., 2009) and in infants (Reid et al., 2007). However, our understanding of infants’ neural representations of others’ actions and intentions is largely based on non-interactive screen-based tasks (e.g., Southgate et al., 2014; Begus et al., 2020). For example, Southgate and colleagues, (2014) explored infants’ sensitivity to changes in the goal of an action sequence during repeated viewing of basic action sequences. These sequences involved an object approaching another object and pushing it around a barrier. The goal was manipulated by altering which object was approached and pushed (one of two). Little is known about how goal representation in the infant brain might be associated with behavioural and neural coordination with caregivers during early social interaction.

Previous research has already shown the utility of event-locked methods for studying mentalisation processes in adults (Cohen et al., 2009) and infants. For example, Goupil and colleagues (2016) used event-locked potentials (ERPs) to examine infants’ capacity to internally monitor the accuracy of their own decision-making. Future research could apply these same methods to further our understanding of the neural substrates of mentalising/ higher-order cognition in early infancy and investigate how these processes are related to behavioural coordination and inter-brain coordination during early social interactions.

## 4 Limitations of event-locked approaches and future approaches

Event-locked analyses are, of course, not without limitation. First, the assumption underlying the principle of averaging brain data relative to behavioural events has been challenged on the basis that it assumes that the averaging process destroys non-phase-locked activity (Mazaheri, 2022). This may be important in instances where event-locked neural signals are not phase-locked (for further discussion see Kalcher and Pfurtscheller, 1995). Second, extracting events from free-flowing naturalistic data can be more challenging than it appears. For example, when excerpting brain activity from 1000msec before a gaze shift to 1000msec afterwards, there may be instances where additional gaze shifts have taken place during that 2000msecs window. However, as long as the degree of artifact is equivalent between the comparison of interest (e.g. where infants lead vs. follow a look to mutual gaze (Fig. 6B)), it is unlikely that differences between events are driven by differences in artifact around those events (Phillips et al., 2023; Marriott Haresign et al., 2023). Future research might also use source separation techniques (see Fig. 7) to examine how mimicry operates via multi-modal pathways – so, for example, combinations of gaze, touch and physical position can influence a child’s eye gaze behaviours in combination, but not alone (Yu and Smith, 2017). To test this, variants of Independent Component Analyses can be used to track co-occurrences across behavioural modalities and separate out multiple overlapping sources of brain activity. This approach would of course necessitate combining information with differing levels of autocorrelation from multiple modalities, making it hard to distinguish reactive from anticipatory changes.

## 5 Summary and conclusions

In this article, we have argued that to understand the mechanisms that drive inter-brain coordination during free-flowing naturalistic interaction we must study fine-grained temporal time dynamics in behaviour. We have argued this on two counts. First, if we do not track how behaviours also vary we cannot be sure about the role that behaviours play in contributing to any neural coordination outcomes, either via artifact or through genuine brain-behavioural associations. Second, we will not be able to better understand the contributions that different modalities and temporal scales have on interpersonal behavioural and neural coordination. Here, we have presented evidence for and argued that approaches that emphasise studying fine-grained temporal dynamics, time-locked to behavioural events should be at the core of future research of interpersonal cognitive neuroscience and inter-brain coordination.

## Declaration of Generative AI and AI-assisted technologies in the writing process

During the preparation of this work the authors used ChatGPT in order to improve readability and language. After using this tool/service, the authors reviewed and edited the content as needed and take full responsibility for the content of the publication.

## Declaration of Competing Interest

The authors declare that they have no known competing financial interests or personal relationships that could have appeared to influence the work reported in this paper.
